# Prenatal Activation of Microglia Induces Delayed Impairment of Glutamatergic Synaptic Function

**DOI:** 10.1371/journal.pone.0002595

**Published:** 2008-07-09

**Authors:** Anne Roumier, Olivier Pascual, Catherine Béchade, Shirley Wakselman, Jean-Christophe Poncer, Eleonore Réal, Antoine Triller, Alain Bessis

**Affiliations:** 1 INSERM, U789, Laboratoire de Biologie Cellulaire de la Synapse, Paris, France; 2 Ecole Normale Supérieure, Département de Biologie, Paris, France; 3 INSERM, U839, Avenir Team ‘Plasticity in Cortical Networks & Epilepsy’, Paris, France; 4 Université Pierre et Marie Curie-Paris 6, IFR83, Paris, France; 5 CNRS, Paris, France; James Cook University, Australia

## Abstract

**Background:**

Epidemiological studies have linked maternal infection during pregnancy to later development of neuropsychiatric disorders in the offspring. In mice, experimental inflammation during embryonic development impairs behavioral and cognitive performances in adulthood. Synaptic dysfunctions may be at the origin of cognitive impairments, however the link between prenatal inflammation and synaptic defects remains to be established.

**Methodology/Principal Findings:**

In this study, we show that prenatal alteration of microglial function, including inflammation, induces delayed synaptic dysfunction in the adult. DAP12 is a microglial signaling protein expressed around birth, mutations of which in the human induces the Nasu-Hakola disease, characterized by early dementia. We presently report that synaptic excitatory currents in mice bearing a loss-of-function mutation in the DAP12 gene (DAP12^KI^ mice) display enhanced relative contribution of AMPA. Furthermore, neurons from DAP12^KI^ P0 pups cultured without microglia develop similar synaptic alterations, suggesting that a prenatal dysfunction of microglia may impact synaptic function in the adult. As we observed that DAP12^KI^ microglia overexpress genes for IL1β, IL6 and NOS2, which are inflammatory proteins, we analyzed the impact of a pharmacologically-induced prenatal inflammation on synaptic function. Maternal injection of lipopolysaccharides induced activation of microglia at birth and alteration of glutamatergic synapses in the adult offspring. Finally, neurons cultured from neonates born to inflamed mothers and cultured without microglia also displayed altered neuronal activity.

**Conclusion/Significance:**

Our results demonstrate that prenatal inflammation is sufficient to induce synaptic alterations with delay. We propose that these alterations triggered by prenatal activation of microglia provide a cellular basis for the neuropsychiatric defects induced by prenatal inflammation.

## Introduction

Epidemiological data suggest that maternal infection during pregnancy increases the risk of neuropsychiatric disorders such as schizophrenia and autism in the offspring [1], [2]. These public health studies are supported by experimental data in rodents showing that maternal inflammation during fetal development impairs the behavior and social interactions of the adult offspring [3]–[5]. The mechanisms that link a transient prenatal inflammation with delayed impairment of neuronal functions have begun to be investigated at the cellular and molecular levels. The symptoms detected in the offspring after injection of inflammatory or infectious agents into pregnant dams are due to the inflammatory response itself, rather than to chemicals, bacteria, or viruses [1], [3]. Recently, in a model mimicking a maternal viral infection, the behavioral alterations in the progeny were prevented by systemic injection of blocking antibodies against interleukin-6 [5]. This reinforces the hypothesis that the deleterious effects arise from the reaction of the immune system, rather that from the inflammatory agent itself. Moreover, several groups have found modifications of protein and mRNA levels of various cytokines and neurotrophic factors in the offspring [4]–[7], suggesting that the immune system of the embryos also contributes to inflammation. The role of microglia in this process has been little addressed [8].

Some cellular bases for impaired brain functions in adult offspring following a prenatal infection have been proposed. For instance, neurogenesis and apoptosis are altered in the adult offspring [4], and morphological damages have been reported [9], [10]. However, whereas it is now accepted that synaptic dysfunctions are responsible for the cognitive impairments observed in psychiatric disorders or neurodegenerative diseases [11], the structure and function of synapses following embryonic inflammation have not yet been investigated. We previously reported a link between immune system impairment and synaptic dysfunction, after having found an enhanced synaptic plasticity and an altered composition of synaptic AMPA receptors in mice mutated on a microglial gene [12]. These mice, DAP12^KI^ (previously referred to as KΔ75 mice [13]) carry a loss-of-function mutation in the gene encoding DAP12, a 12 kD transmembrane signaling adaptor for a family of innate immunoreceptors. In the immune system, DAP12 regulates the activation of peritoneal or bone-marrow-derived macrophages [14]–[16]. In the brain, DAP12 is transiently expressed by developing microglia [12], and its expression can be reinduced in adult upon pathological conditions [17]–[19]. In culture, DAP12 also controls the phagocytic activity of microglia and the inflammatory response following neuronal death [20]. In humans, DAP12 mutations induce Nasu-Hakola disease (OMIM 221770), characterized by early dementia without any apparent neuronal damage [21].

Microglia are the instrumental cells of brain inflammation, therefore we investigated here the relationship between prenatal activation of microglia and alteration of synaptic function. We first demonstrated that the ratio between AMPA and NMDA receptor (AMPAR, NMDAR) currents is increased in adult DAP12^KI^ mice, as compared to wild-type (WT). Then, by analyzing pure neuronal cultures from DAP12^KI^ P0 pups, we investigated the impact of the prenatal period in inducing late synaptic defects. Finally, as we observed that DAP12^KI^ animals displayed a transient microglial activation at birth, we compared their synaptic phenotype with the one of WT animals having experienced a pharmacologically-induced inflammation during their fetal development. We conclude from both genetical and pharmacological models that prenatal activation of microglia has a delayed impact on synaptic function.

## Results

### Mutation of the microglial protein DAP12 enhances relative AMPAR contribution to glutamatergic transmission in the hippocampus

**Figure 1:**
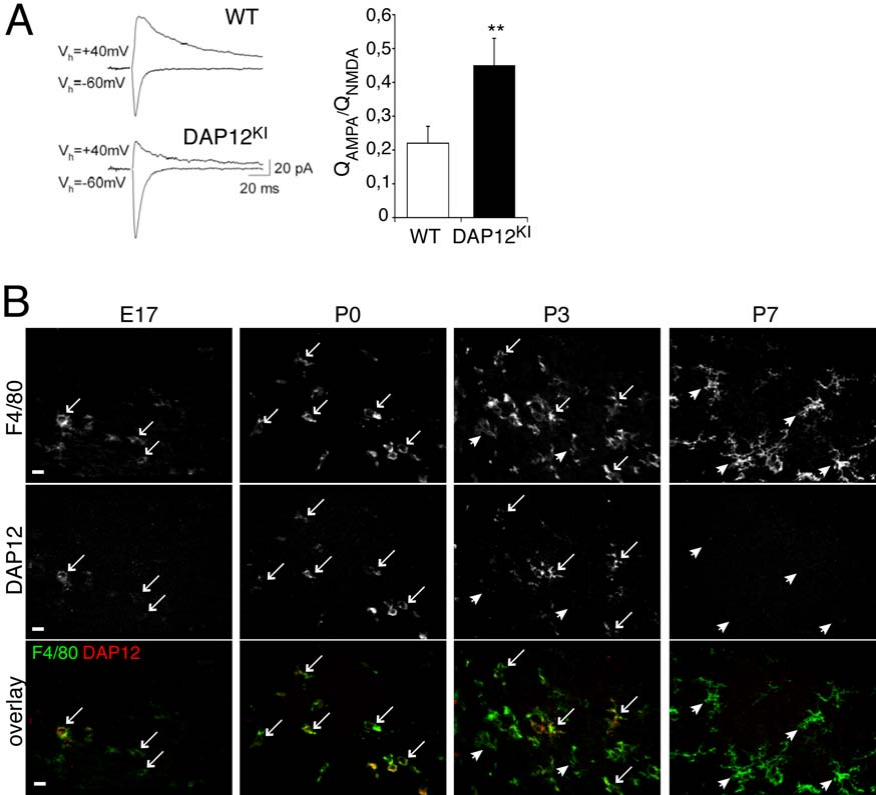
DAP12 mutation induces an increased ratio of AMPAR vs. NMDAR currents in adult hippocampal slices, whereas DAP12 is expressed only by microglia around birth. (A) Ratio of AMPAR vs. NMDAR currents in hippocampal slices. Left, original traces from individual experiments. Right, averaged data from 9 and 10 recordings of WT and DAP12^KI^ mice, respectively, Mean±SEM, **p<0.03, Mann-Whitney t test. (B) Double detection of DAP12 (upper line), of the microglial marker F4-80 (middle line), and overlay of both stainings (lower line), in the developing hippocampus. DAP12 immunoreactivity is restricted to F4-80 positive cells. The fraction of microglia that express DAP12 (arrows: DAP12-positive microglia) culminates at P0, when microglia are ameboid or poorly ramified. Postnatally, when microglia become ramified, they express less DAP12 and the fraction of DAP12-negative microglia (arrowheads) increases. At P7, very few DAP12-positive microglia are found (none in this picture). Scale bar: 10 µm.

We previously reported that in the synapses of the Schaffer collaterals onto CA1 pyramidal neurons of DAP12^KI^ mice, the NMDA receptors (NMDARs) were more sensitive to the NR2B-specific antagonist ifenprodil, and the AMPARs were more permeable to calcium, as compared with WT mice [12]. Noteworthy, in synapses formed by dentate gyrus granule cells onto interneurons, it has been shown that Ca^2+^-permeable AMPARs and high content of NR2B in NMDAR result in an enhanced contribution of AMPAR to the glutamatergic response, with high ratio of AMPAR- versus NMDAR-evoked excitatory post-synaptic currents (EPSC) [22]. Thus, in order to further understand the consequence of DAP12 mutation on synaptic function, we have now addressed the relative contribution of AMPAR vs. NMDAR EPSCs in the Schaffer collateral synapses formed onto CA1 pyramidal neurons in DAP12^KI^ mice. In WT mice, the ratio of AMPAR- over NMDAR-mediated currents was 0.23±0.05 (n = 9), while in DAP12^KI^ mice, this ratio was enhanced ∼2-fold (Fig 1A; DAP12^KI^: 0.45±0.08; n = 10; p<0.03). Thus, the relative contribution of AMPAR vs. NMDAR to glutamatergic currents is increased in the hippocampus of mutant mice. With electron microscopy analysis, we found that the synaptic density was unchanged in the hippocampus of DAP12^KI^ mice (supplementary data, Fig S1B), but the density of perforated synapses was significantly increased in the stratum radiatum of their CA1 area (supplementary data, Fig S1CD). This is consistent with the enhanced AMPAR/NMDAR ratio in DAP12^KI^ hippocampus, since perforated synapses are more enriched in AMPARs than in NMDARs [23].

### Mutation of the microglial protein DAP12 has a delayed impact on synaptic function

**Figure 2:**
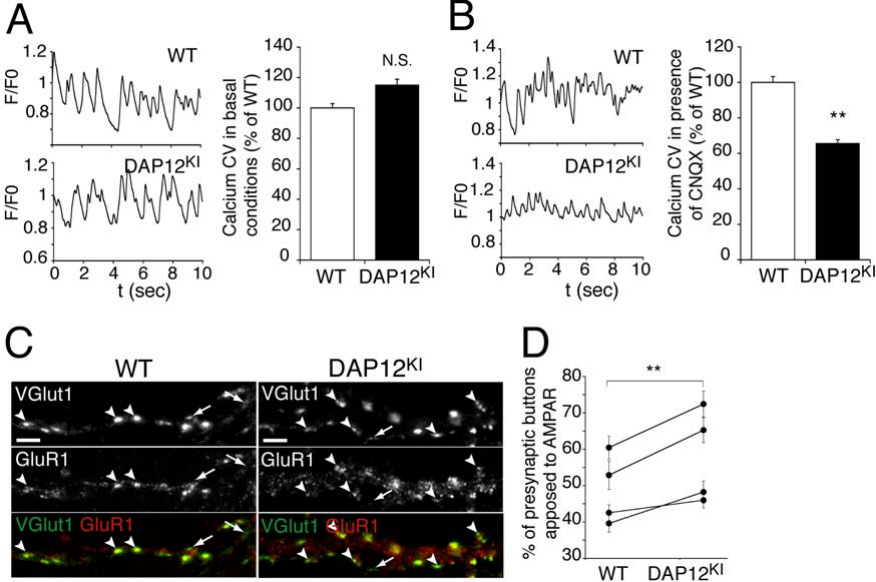
Neurons from DAP12^KI^ P0 pups cultured without microglia develop synaptic alterations. (A) Coefficients of variation (CV, see Methods), in basal conditions, of the fluctuations of intracellular calcium concentration, in neurons cultured from WT and DAP12^KI^ animals. Left, original recordings from individual neurites; right, averaged data from three independent experiments (>200 neurites recorded/experiment). (B) Coefficients of variation in presence of CNQX, an AMPAR antagonist. Left, original recordings from individual neurites; right, averaged data from three independent experiments (>200 neurites recorded/experiment); **p<0.0001 Mann-Whitney t test. (C) Double detection of VGlut1 (green or upper line) and GluR1 (red or middle line) immunoreactivity, and overlay of both stainings (lower line) in neuronal cultures from WT and DAP12^KI^ animals. Arrowheads: apposition of VGlut1 and GluR1 puncta; arrows: VGlut-1 puncta not apposed to GluR1 clusters. Scale bar: 5 µm. (D) Quantitative analysis of VGlut-1 and GluR1 clusters association in four pairs of cultures from WT and DAP12^KI^ pups. At least 40 dendrites were analyzed for each culture. **p<0.0001, two-way ANOVA.

The above-described results raise a temporal paradox. DAP12 mutation impacts glutamatergic transmission in the adult, but we previously demonstrated that it ceases to be expressed in the hippocampus at this age [12]. To solve this paradox, we examined more precisely the expression pattern of DAP12 in developing hippocampus of WT mice (Fig 1B). At E17, microglia were sparse and mostly ameboid. A majority displayed DAP12 immunoreactivity, albeit at low level. At P0, the density of microglia increased, and most displayed a bright immunostaining for DAP12. Between P0 and P3, the microglia became ramified and the fraction of DAP12-expressing microglia decreased. At P7, microglia were mostly ramified, and few DAP12-positive microglia were detected. Given that DAP12 is mainly expressed before P3 and that glutamatergic synapses are generated post-natally [24], we hypothesized that microglial dysfunction induced by DAP12 mutation has impacted the prenatal development of neurons with delayed consequences on synaptic function. To test this hypothesis, we cultured neurons from DAP12^KI^ and WT littermate P0 pups in the presence of AraC, so as to eliminate microglia. Neurons were grown for at least two weeks in culture to allow maturation of glutamatergic synapses [25]. At that point, neuronal cultures were more than 99% pure (supplementary data, Fig S2). Fluorometric calcium imaging was used to monitor neuronal activity. Since spontaneous activity was moderate in hippocampal cultures, all recordings were performed in the presence of the potassium channel blocker 4AP (50 µM) to increase the global network activity. Under these conditions, neurons cultured from WT and DAP12^KI^ P0 displayed similar activity (coefficient of variation of calcium fluctuation in DAP12^KI^ cultures: 115±4% of WT; Fig 2A). Yet, in the presence of 6-cyano-7 nitroquinoxline-2 (CNQX), an AMPAR antagonist, neuronal activity was significantly lower in neurons cultured from mutant as compared with WT littermates (coefficient of variation of calcium fluctuations in DAP12^KI^ cultures: 66±2% of WT; n>600; p<0,0001; Fig 2B). These results are consistent with the enhanced AMPAR/NMDAR currents ratio observed *in vivo*. They indicate that the contribution of AMPARs to neuronal activity is increased in DAP12^KI^ neurons cultured in the absence of microglia. This increased contribution of AMPAR may possibly reflect an increased proportion of excitatory synapses containing AMPA receptors. To test this, glutamatergic synapses and AMPAR were identified by vesicular glutamate transporter 1 (VGlut1) and by GluR1 immunoreactivities, respectively (Fig 2C). Figure 2D shows that the proportion of excitatory synapses colocalized with GluR1-positive puncta was significantly increased in cultures from mutant pups as compared with WT pups (n>160; p<0.0001). In conclusion, neurons from DAP12^KI^ P0 pups cultured in the absence of microglia exhibit an enhanced relative contribution of AMPAR to excitatory transmission, which is compatible with the synaptic phenotype observed *in vivo*. This demonstrates that microglial alteration due to DAP12 loss-of-function impacts neurons before birth, with delayed effects on synaptic function.

### DAP12 mutated microglia display a partial inflammatory status

**Figure 3:**
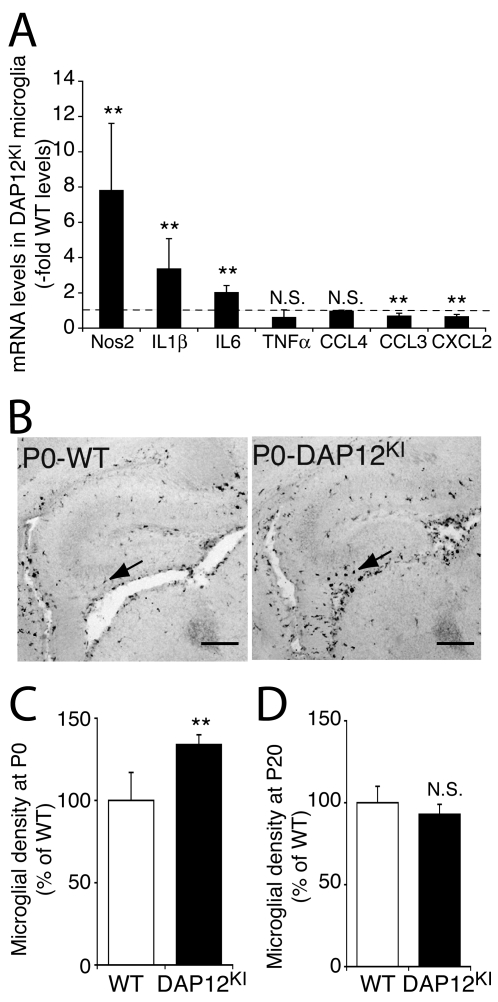
Microglia of DAP12^KI^ mice display a partially activated phenotype at birth. (A) Quantitative analysis of the expression of inflammatory markers in DAP12^KI^ microglia. Quantitative RT-PCR performed on pure microglia cultured from brains of WT or DAP12^KI^ P0 pups. The levels in the mutant microglial cultures are expressed relative to the levels in the WT microglial cultures. n = 3 pairs of cultures. **p<0.05, Student's t test. (B) F4/80 labeling of microglia (arrows) in the hippocampus of WT and DAP12^KI^ pups. Scale bar: 100 µm. (C) Quantification of microglial density in the hippocampus of WT and DAP12^KI^ animals at P0. Between 4 and 15 pairs of matched slices were counted per experiment, n = 3 experiments; **p<0.001, two-way ANOVA. (D) Quantification of microglial density (Iba-1 positive cells) at P20 (n = 4 experiments).

In order to understand how neurons from DAP12^KI^ mice are perturbed prenatally, we characterized the effects of DAP12 loss-of-function on the expression level of microglial genes encoding proteins known to modulate synaptic or neuronal functions. As shown in figure 3A, microglia cultured from DAP12^KI^ neonates expressed higher levels of the mRNAs of the NO synthesizing enzyme NOS2, and of the cytokines interleukin-1β and -6 (IL1β, IL6), all of which are inflammatory markers. The mRNA levels of the chemokines CCL3 and CXCL2 slightly decreased (Fig 3A; n = 3; p<0.05). Finally, no change was observed in the expression level of TNFα, another inflammatory marker, which regulates AMPAR trafficking to synapses [26], [27].

The overexpression of some, but not all inflammatory markers, indicates that DAP12 loss-of-function induces a partial activation phenotype in microglial cells. Inflammation is characterized by expression of specific markers as well as increased proliferation. To further characterize the inflammation status resulting from DAP12 mutation, we quantified the density of microglia in the hippocampus of DAP12^KI^ and WT P0. We found that microglial density was enhanced in the hippocampus of DAP12^KI^ P0 pups as compared with WT at P0 (134±6% of WT; p<0.001; Fig 3BC). In the adult brain, however, the microglial cell density was not different in DAP12^KI^ as compared with WT mice (DAP12^KI^: 98±12% of WT, Fig 3D). These data indicate that a partial and transient inflammation occurs around birth in DAP12^KI^ mice. This prenatal activation of microglia could thus be involved in the delayed impaired synaptic function of DAP12^KI^ mice.

### LPS-induced prenatal activation of microglia results in delayed synaptic alterations

To test whether fetal activation of microglia could be sufficient to induce delayed synaptic defects, we induced prenatal pharmacological inflammation and evaluated the consequences on synaptic function.

**Figure 4:**
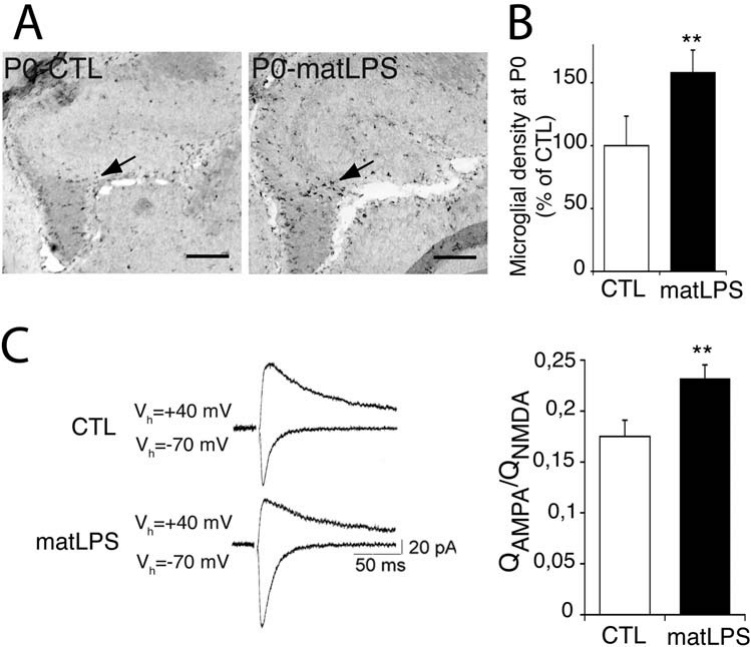
Maternal exposure to LPS induces microglial activation at birth and synaptic alterations in the adult offspring. (A) F4/80 labeling of microglia (arrows) in the hippocampus of P0 born from control (CTL) or LPS-injected dams (matLPS). (B) Quantification of microglial density in the hippocampus at P0. Between 4 and 15 pairs of matched slices were counted per experiment, n = 4 experiments; **p<0.001, two-way ANOVA. Scale bar: 100 µm. (C) Increased ratio of excitatory AMPAR and NMDAR currents in hippocampal slices of adults born to control (CTL) or LPS-injected (matLPS) dams. Left, original traces from individual experiments. Right, averaged data from n = 9 CTL and 10 matLPS experiments; **p<0.02.

In mice, the generation of hippocampal neurons starts at around E14 [28], and microglia begin to invade parenchyma at around E15 [29]. We thus choose to induce an inflammation at E15. We induced inflammation by injecting a low dose (0,12 mg/kg) of *E. coli* lipopolysaccharide (LPS) intraperitoneally into pregnant dams. Low doses of lipopolysaccharide do not cross the placental barrier [30]. Therefore, the fetal inflammation is not mediated by the injected agent itself, but rather by the maternal immune response, possibly in coordination with fetal immune cells [1], [3], [8], [31]. This treatment did not alter the birth date or the size of the litters (n = 12±4 and 11±6 living pups per control and LPS-injected dams, respectively; p = 0.62, Student's t test). To check whether this treatment mimicked the inflammation in DAP12^KI^ pups, we analyzed the microglial density in the offspring born to LPS-injected mother. As shown in Figure 4B, microglial density was increased in the hippocampus of P0 born to LPS-injected dams (158±18% of control; n = 37 pairs of slices; p<0.001; Fig 4AB). This increase was comparable with the one observed in DAP12^KI^ pups (see above). We next evaluated the consequence of such prenatal inflammation on glutamatergic transmission. To do so, we first measured the ratio of AMPAR versus NMDAR EPSCs in an adult born to inflamed mother. As in DAP12^KI^ mice, the relative contribution of AMPAR to evoked EPSCs was enhanced in hippocampal slices taken from adult mice that had been subjected to fetal inflammation (AMPAR/NMDAR currents ratio: Control: 0.18±0.02 (n = 9); LPS-treated mice: 0.23±0.01 (n = 10); p<0.02; Fig 4C).

**Figure 5:**
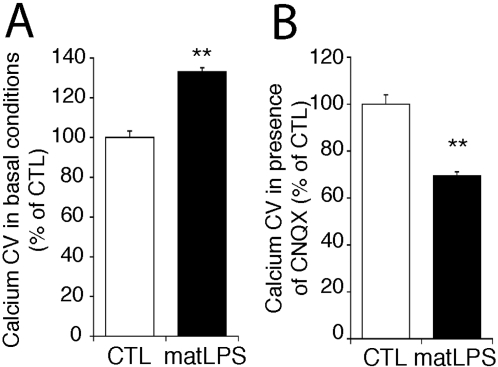
Pharmacological activation of microglia during embryonic development induces late synaptic alterations in culture. (A) Coefficient of variation (CV, see Methods), in basal conditions, of the fluctuations of intracellular calcium concentration, in neurons cultured from pups born from control (CTL) or LPS-injected (matLPS) dams. Averaged data from three independent experiments (>200 neurites recorded/experiment); **p<0.0001 Mann-Whitney t test. (B) Coefficient of variation in the presence of AMPAR antagonist CNQX. Averaged data from three independent experiments (>200 neurites recorded/experiment) **p<0.0001 Mann-Whitney t test.

In order to evaluate whether LPS-induced prenatal inflammation also impairs synaptic function with delay, we analyzed the neuronal activity of neurons cultured from P0 pups born to control and to LPS-injected dams, both in the absence of microglia. In basal conditions, neuronal activity, monitored by calcium concentration fluctuations, was higher in neurons from maternally inflamed P0, as compared with controls (134±4% of control; n>600; p<0.0001; Fig 5A). Yet, calcium fluctuations in the presence of CNQX were significantly lower in neurons from maternally inflamed P0 than from controls (69±2% of control; n>600; p<0.0001; Fig 5B). This resembles what we observed in neurons cultured from DAP12^KI^ hippocampus. These results demonstrate that prenatal activation of microglia, when also done by pharmacological means, is sufficient to induce a delayed increase in the contribution of AMPAR to excitatory neurotransmission.

## Discussion

In this work, we show that prenatal activation of microglia, when both genetically and pharmacologically induced, has a delayed impact on glutamatergic synaptic function in adult.

We previously showed that mutation of the microglial gene DAP12 induced an enhanced long-term potentiation (LTP) and modifications of glutamate receptors at synapses formed onto CA1 pyramidal cells [12]. In particular, we showed that these synapses displayed a somehow immature phenotype, in which the NMDARs were more sensitive to the NR2B-specific antagonist ifenprodil, and the AMPARs more permeable to calcium as compared with WT mice [12]. We have now observed that in DAP12^KI^ mice, these synapses also display a higher ratio of AMPAR versus NMDAR EPSC. Such a correlation between AMPAR/NMDAR composition and properties and AMPAR/NMDAR currents ratio has been reported in another kind of synapses [22]. In DAP12^KI^ mice, the increased AMPAR to NMDAR currents ratio may also synergistically result from the increased density of perforated synapses that we measured, since these synapses are especially enriched with AMPARs [23], [32]. This increased density was restricted to the medial segment of the apical dendrites of CA1 neurons and may contribute to the EPSCs that we monitored [33], [34].

We previously proposed that the enhanced LTP in DAP12^KI^ mice may be due to a larger fraction of silent, AMPAR-less, synapses in mutant mice. Such hypothesis was based on biochemical analysis of post-synaptic densities (PSD) that showed a decreased content of AMPAR in DAP12^KI^ mice brain as compared with WT. We did not observe this decrease in our present analysis of hippocampal cultures and hippocampal synapses morphology. This apparent discrepancy probably results from the fact that in order to get an adequate amount of material, the prior PSD analysis had been performed on whole cortex extracts, and this may have occluded subtle modifications of synaptic AMPARs restricted to the more specific regions under investigation in our current study. Moreover, recent reports in the literature have demonstrated that switching synaptic NR2B-containing NMDARs with those containing NR2A, dramatically reduces LTP [35]. Indeed, in DAP12^KI^ hippocampus, NMDAR were sensitive to the NR2B-specific antagonist ifenprodil [12]. Thus, the enhanced LTP previously observed in DAP12^KI^ hippocampus may be the consequence of the modifications of NMDARs. In order to get an insight into the mechanism linking DAP12 loss-of-function and neuronal phenotype, we characterized the microglial alterations induced by the mutation. We showed that DAP12^KI^ microglia cultured from P0 pups express higher levels of inflammatory markers such as NOS-2, IL1β, and IL6, as compared with their WT counterparts. The inflammatory status of DAP12^KI^ was further confirmed by the detection of a transient microgliosis in new-born, but not in adult mice brains. It has recently been demonstrated that in peripheral macrophages, DAP12 signaling is activator or inhibitor depending on the inflammatory context [14], [15]. It has been proposed that upon a strong inflammatory stimulus such as a high level of LPS, DAP12 participates in activatory pathways, whereas at a low level of LPS, DAP12 may preferentially recruit inhibitory mediators [14]. Our data suggest that in the developing brain, DAP12 is a down-regulator of microglial activation.

Neurons cultured from DAP12^KI^ or inflamed P0 pups that were grown in a microglia-free and non-inflammatory environment display altered synaptic function. This demonstrates that prenatal inflammation suffices to alter synaptic function. However, this does not exclude the possibility that prenatal inflammation also has long-lasting effects on tissue and secondarily impacts neuronal or synaptic dysfunction. For instance, adult mice born to inflamed mother can display histological alterations [9], [10] or impaired neurogenesis [4], which may result in disturbance of neurotransmission. Similarly, old DAP12-deficient mice display hypomyelinosis in thalamus [36], [37]. Such deficiencies demonstrate persistent changes that we have not addressed in the present study. Together with white matter alteration, a synaptic degeneration has been described in the thalamus of DAP12-deficient mice, based on abnormal vesicles accumulations observed by electron microscopy [36]. We did not detect morphological alterations of synapses in the hippocampus of DAP12^KI^ by electron microscopy (supplementary data, Fig S1 A). Such a difference between the two studies may be due to differences in age, in the genetic background of the mice, or in the brain regions studied. Alternatively, the difference may arise from the fact that the vesicle accumulations, described as being a sign of synaptic degeneration [36], were not located in synapses as defined by the apposition of vesicles, presynaptic dense projections, synaptic cleft, and post-synaptic density [38]. Rather, these accumulations may correspond to features that have been already described and are unrelated to synaptic dysfunction [38].

Prenatal inflammation is known to differentially impact adult neuronal functions, depending on the inflammation-inducing protocol [4]. In our study, we found that neurons cultured from maternally-inflamed pups display enhanced basal neuronal activity as compared with control. In contrast, basal activity was identical in neurons from DAP12^KI^ and WT pups. This shows that the genetical and pharmacological inductions of prenatal microglial activation induce similar but not identical synaptic phenotypes. Such a difference may be due to TNFα, whose mRNA is not upregulated in DAP12^KI^ microglia, but which is a hallmark of inflammation [4].

We have now shown that activation of microglia impacts neurons prenatally, with a delayed effect on their synaptic function. Deferred alteration of synaptic function depending on prenatal development has already been observed. For example, synaptic activity can be recorded on neurons from E18 rat hippocampus cultured for two weeks, whereas it is barely detected when neurons are cultured from E16 embryos [39]. This indicates that prenatal differentiation of neurons is crucial for the later development of synaptic function.

Epidemiological data have shown that infection during pregnancy increases the risk of schizophrenia and autism in adulthood [2], [40]. In humans, mutations of DAP12 induce the Nasu-Hakola disease, a presenile dementia that occurs in subjects in their 30's. Adult mice knocked-out for DAP12 and adult mice born to inflamed mothers display behavioral or cognitive deficits reminiscent to some of the symptoms of autism and shizophrenia [3], [4], [36]. Dementia are now mostly described as synaptopathies [11], and alterations of glutamatergic synapses have been involved in the etiology of autism [41]. In addition, hypofunction of NMDAR has been implicated in the physiopathology of schizophrenia [42], [43]. Without excluding the involvement of secondary histological alterations, the data presented in this work provide a cellular basis for the neuropsychiatric defects induced by prenatal inflammation.

## Materials and Methods

### Animals and LPS injection

DAP12^KI^ mice, also known as KΔ75 mice [13] are knocked-in with an allele bearing a loss-of-function mutation in DAP12/KARAP gene. They were backcrossed eight times with C57Bl/6J/Rj mice. For each experiment, we used DAP12^KI^ and WT littermates. Lipopolysaccharide (LPS, *E. coli* 055∶B5; Sigma, France) was injected intraperitoneally into pregnant Swiss mice at 0.12 mg/kg, at day 15 of gestation. PBS injection was used as a control. The experimental procedures were approved by the Animal Experimentation Regional Ethics Committee in (p3-2007-011 and p3-2005-012).

### Cultures and quantitative RT-PCR

For neuronal cultures, hippocampi from P0 animals were dissociated by trituration after incubation with papain. Cells were grown in astrocyte-conditioned medium supplemented with 5 µg/ml AraC (Calbiochem, France) to eliminate microglia and astrocytes. Efficiency of AraC treatment was controlled both by immunofluorescent stainings of cultures for the microglia-specific F4/80 and astrocyte-specific GFAP markers, and by RT-PCR for GFAP, Mac1 and NR1. Immunofluorescence showed that neuronal cultures contained less than 0,7% of total glial cells after 8 days of culture (see Fig S2 for details). To minimize variations attributable to potential fluctuations in culture conditions, we compared neurons from DAP12^KI^ or WT and from maternally inflamed or control P0 pups in sister cultures. For astrocytes cultures, cortices from P1 Swiss pups were dissociated by trituration after incubation with trypsin. Cells were grown in MEM supplemented with horse serum. When confluence was reached, this medium was replaced with Neurobasal medium supplemented with B27 (Invitrogen, France). Three times a week, this astrocyte-conditioned medium was removed, filtered and fed to neurons, and replaced with fresh Neurobasal plus B27.

Microglia were cultured from P0 cortices and grown in DMEM (Invitrogen, France) with FCS (BioWest, France). Microglia were detached from the astrocytic layer after 12 days *in vitro* by gentle shaking, settled on new plastic dishes, and processed after 24 hrs for RNA purification (Ambion Europe, UK).

RNA was extracted using the MicroRNAqueous kit (Ambion Europe, UK), treated with DNase and reverse-transcribed with SuperScript II (Invitrogen, France). Quantitative PCR were performed with the QuantiTect SYBR Green PCR kit (Qiagen, France) on a LightCycler (Roche Diagnostics GmbH, Germany). 7SK RNA content was used for normalization. All primers were designed with Primer3 [44].

### Electrophysiology

Hippocampal slices were prepared from 18-25-day old WT (n = 14) and DAP12^KI^ (n = 13) mice or from mice born to LPS (n = 9) or control PBS-injected (n = 10) mothers, as described in [12]. Whole-cell recordings were obtained from CA1 pyramidal cells using borosilicate glass microelectrodes (2–5 MΩ) containing 115 mM CsMeSO_3_, 20 mM CsCl, 10 mM HEPES, 0.1 mM EGTA, 4 mM Mg-ATP, 0.4 mM Na_3_-GTP and 10 mM Na-phosphocreatine. EPSCs were evoked by extracellular stimulation of Schaffer collateral afferents in the presence of 20 µM bicuculline methochloride after a cut was made between the CA3 and CA1 areas. AMPA- and NMDA-receptor mediated currents were measured from recordings made at −60 and +40 mV, respectively (−70 and +40 mV in offspring of control and LPS-injected mice). In order to compare AMPA/NMDA currents ratios, stimulation intensities were adjusted to induce EPSCs of similar amplitudes when recorded at −60 (or −70) mV.

### Calcium imaging

Neurons grown for 20 days were loaded with Fluo4-AM (Molecular Probes, France). Recordings (one image every 100 ms for 10 seconds) were made on neurites in the presence of 50 µM potassium channel blocker 4AP (Sigma Aldrich, Lyon, France) and possibly CNQX (50 µM, Tocris Cookson, Bristol, UK). Changes in the fluorescence level (F), normalized to the basal fluorescence at the beginning of the recordings (F0), reflected intracellular calcium fluctuations. The coefficients of variation (CV = standard deviation/mean) of F/F0 were calculated with TI Workbench Software (kindly provided by Takafumi Inoue, Waseda University, Tokyo, Japan).

### Immunostaining

Brain sections were obtained and processed as described in [12], except for E17 embryo brains that were fixed by immersion in 4% paraformaldehyde.

For immunohistochemistry, rat anti-F4/80 (Serotec, Oxford UK) and rabbit anti-DAP12 (Chemicon, UK) primary antibodies were incubated for 48 hrs at 4°C, then revealed by goat anti-rat-Alexa488 (Molecular Probes, France) and donkey anti-rabbit-Cy3 (Jackson Immunoresearch Laboratories, USA) antibodies, respectively.

For immunoperoxidase stainings, rat anti-F4/80 (Serotec, Oxford UK) and rabbit anti-Iba-1 (Wako, Osaka, Japan) were used as primary antibodies to label microglia at P0 and P20, respectively; biotinylated anti-rat and anti-rabbit (Amersham Health SA, France) were used as secondary antibodies, respectively. Sections were revealed with avidin-biotin complex (Vectastain ABC elite, Vector Laboratories, UK) and diaminobenzidine. Only paired slices of DAP12^KI^/WT, or CTL/LPS animals (same position in hippocampus) were selected for analysis. At least six pairs of slices were analyzed per experiment.

Cultured neurons were processed as described [12]. Primary antibodies were guinea pig anti-VGlut1 and rabbit anti-GluR1 (both from Chemicon, UK), secondary antibodies were goat anti-guinea pig-FITC and goat anti-rabbit-Cy3 (both from Jackson Laboratories, West Grove, PA). Quantifications were performed as described in [45].

## Supporting Information

Figure S1 Increased density of perforated synapses in DAP12^KI^ hippocampus. (A) Synapses, defined by the apposition of presynaptic vesicles (v), synaptic cleft (c) and post-synaptic density (p) were not different between WT and DAP12^KI^. In particular, no signs of synaptic degeneration. (B) Synaptic density in WT and DAP12^KI^ hippocampus, measured in the stratum radiatum of the hippocampus, at 50 to 200 µm of the pyramidal cell layer. 683+/−61 (WT) and 68+/−18 (DAP12^KI^) synapses were counted per animal. Results are mean+/−SD (n = 3, p = 0.33 t-test). (C) A perforated synapse (double arrow) defined by a single bouton (v) apposed with two post-synaptic densities (p). Note that the membranes are collapsed between the post-synaptic densities. (D) Quantification of the percentage of multiple synapses at 150 to 200 µm of the pyramidal cell layer. Results are mean±SD (n = 3, **p = 0.004; t-test). No differences were observed from 50 to 150 µm (not shown). Scale bars = 0.2 µm.
(8.45 MB TIF)

Figure S2 Purity of hippocampal neuronal cultures treated with AraC. A,B: Percentages of: (A) astrocytes (GFAP-positive), (B) microglial cells (F4/80-positive), in neuronal cultures fixed after 2, 8 or 15 days of culture in presence of AraC. The total number of cells was assessed with DAPI staining. n = 150 to 550 cells were counted per condition. Means±SD from 2 cultures are presented. At 15 DIV, we counted 350 cells without finding any microglia, indicating that the percentage of microglial cells was less than 0,3%. C: RT-PCR on neuronal (2, 8 or 13 days-old), astrocytic (“Astro.”) or microglial (“Mg.”) cultures. “-RT”: template without reverse-transcriptase. Each PCR was performed on cDNA corresponding to 3 ng of ARN. 30 cycles were used to amplify the cDNA of: NR1 (NMDAR1, neuronal marker), GFAP (glial fibrillary acidic protein, astrocytic marker), Iba1 (ionized calcium binding adapter molecule 1, microglial marker). GFAP is barely detected in 8 and 13 days-old neuronal cultures, whereas it is detected in 2 days-old neuronal and in astrocytic cultures (positive control). Iba1 is not detected in neuronal cultures, but is expressed in the microglial culture (positive control).
(0.79 MB TIF)
