# Supplementary figures and images for: Prenatal Activation of Microglia Induces Delayed Impairment of Glutamatergic Synaptic Function
Source: PLoS One. 2008 Jul 9;3(7):e2595. doi: 10.1371/journal.pone.0002595 (PMC2440505; doi:10.1371/journal.pone.0002595)

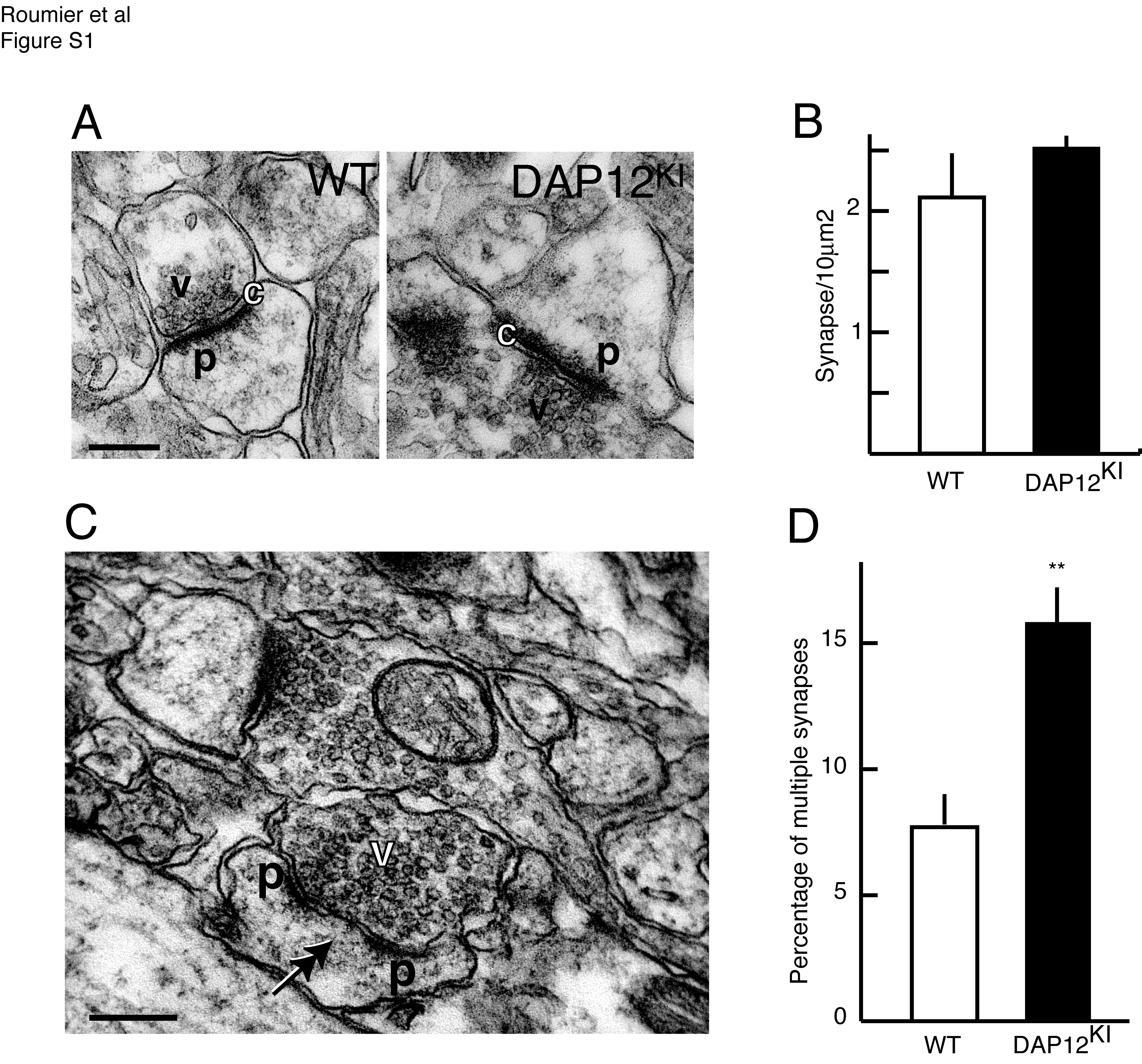

Supplement: Figure S1 — Increased density of perforated synapses in DAP12KI hippocampus. (A) Synapses, defined by the apposition of presynaptic vesicles (v), synaptic cleft (c) and post-synaptic density (p) were not different between WT and DAP12KI. In particular, no signs of synaptic degeneration. (B) Synaptic density in WT and DAP12KI hippocampus, measured in the stratum radiatum of the hippocampus, at 50 to 200 µm of the pyramidal cell layer. 683+/−61 (WT) and 68+/−18 (DAP12KI) synapses were counted per animal. Results are mean+/−SD (n = 3, p = 0.33 t-test). (C) A perforated synapse (double arrow) defined by a single bouton (v) apposed with two post-synaptic densities (p). Note that the membranes are collapsed between the post-synaptic densities. (D) Quantification of the percentage of multiple synapses at 150 to 200 µm of the pyramidal cell layer. Results are mean±SD (n = 3, **p = 0.004; t-test). No differences were observed from 50 to 150 µm (not shown). Scale bars = 0.2 µm. (8.45 MB TIF) [file pone.0002595.s001.tif]

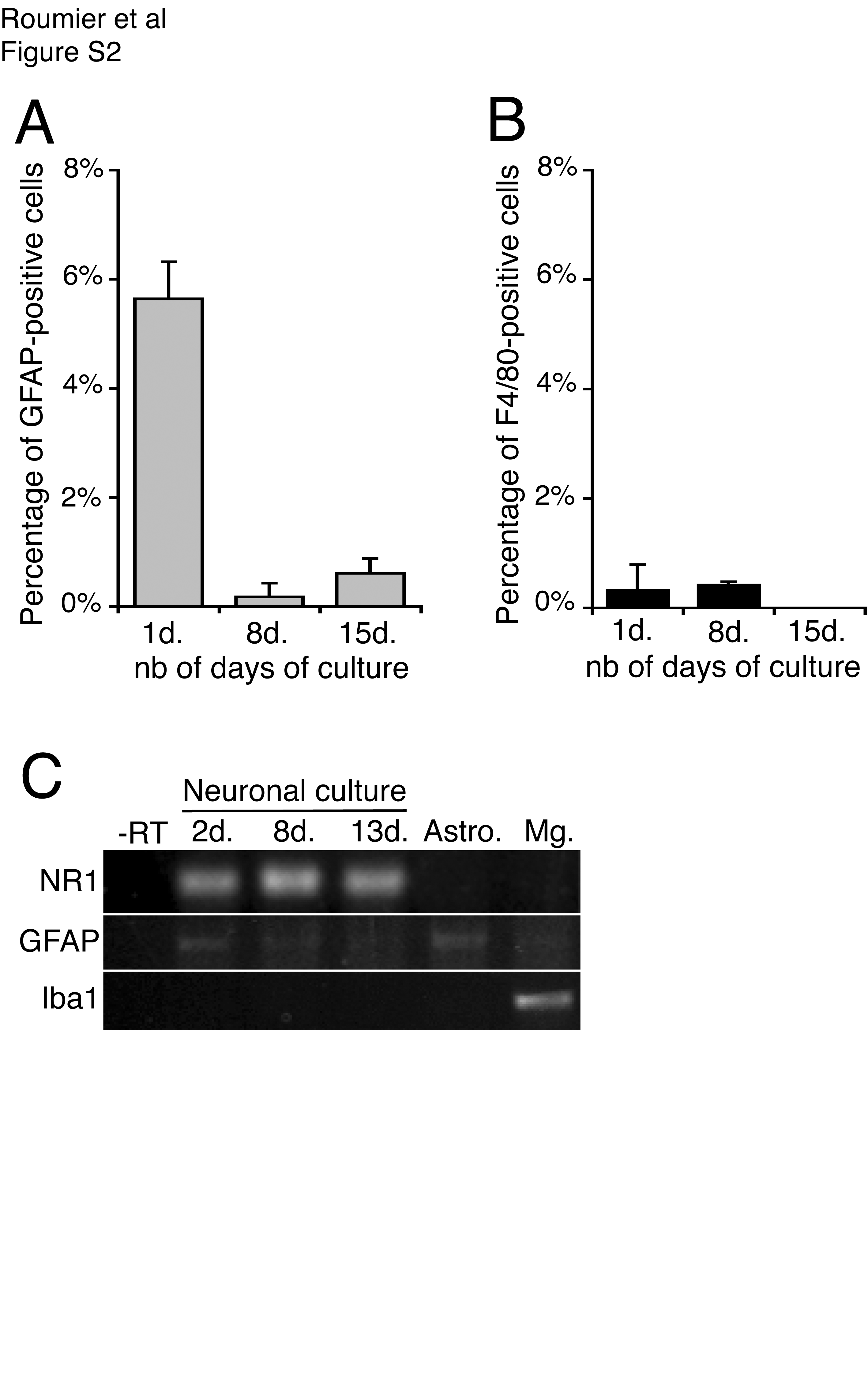

Supplement: Figure S2 — Purity of hippocampal neuronal cultures treated with AraC. A,B: Percentages of: (A) astrocytes (GFAP-positive), (B) microglial cells (F4/80-positive), in neuronal cultures fixed after 2, 8 or 15 days of culture in presence of AraC. The total number of cells was assessed with DAPI staining. n = 150 to 550 cells were counted per condition. Means±SD from 2 cultures are presented. At 15 DIV, we counted 350 cells without finding any microglia, indicating that the percentage of microglial cells was less than 0,3%. C: RT-PCR on neuronal (2, 8 or 13 days-old), astrocytic (“Astro.”) or microglial (“Mg.”) cultures. “-RT”: template without reverse-transcriptase. Each PCR was performed on cDNA corresponding to 3 ng of ARN. 30 cycles were used to amplify the cDNA of: NR1 (NMDAR1, neuronal marker), GFAP (glial fibrillary acidic protein, astrocytic marker), Iba1 (ionized calcium binding adapter molecule 1, microglial marker). GFAP is barely detected in 8 and 13 days-old neuronal cultures, whereas it is detected in 2 days-old neuronal and in astrocytic cultures (positive control). Iba1 is not detected in neuronal cultures, but is expressed in the microglial culture (positive control). (0.79 MB TIF) [file pone.0002595.s002.tif]
